# A label-free quantification method for assessing sex from modern and ancient bovine tooth enamel

**DOI:** 10.1038/s41598-024-68603-4

**Published:** 2024-08-06

**Authors:** Paula Kotli, David Morgenstern, Fanny Bocquentin, Hamoudi Khalaily, Liora Kolska Horwitz, Elisabetta Boaretto

**Affiliations:** 1 Scientific Archaeology and D-REAMS Radiocarbon Dating Laboratory, Weizmann Institute of Science, 760001 Rehovot, Israel; 2 Nancy and Stephen Grand Israel National Center for Personalized Medicine G-INCPM, Weizmann Institute of Science, 760001 Rehovot, Israel; 3 CNRS, UMR 8068 TEMPS, MSH Mondes-Bâtiment Ginouvès, 21 allée de l’université, 92023 Nanterre Cedex, France; 4 Israel Antiquities Authority POB 586, 91004 Jerusalem, Israel; 5 National Natural History Collections, E. Safra-Givat Ram Campus, The Hebrew University of Jerusalem, 96194 Jerusalem, Israel

**Keywords:** Paleoproteomics, Bovine, Zooarchaeology, Peptidomics, Amelogenin, Diagenesis, Enamel, Post-translational modifications (PTMs), Peptides, Archaeology, Proteomics, Computational biology and bioinformatics, Protein analysis, Protein sequence analyses

## Abstract

Identification of the sex of modern, fossil and archaeological animal remains offers many insights into their demography, mortality profiles and domestication pathways. However, due to many-factors, sex determination of osteological remains is often problematic. To overcome this, we have developed an innovative protocol to determine an animal’s sex from tooth enamel, by applying label-free quantification (LFQ) of two unique AmelY peptides ‘**L**R**Y**PYP’ (AmelY;[M+2]$^{2+}$ 404.7212 m/z) and ‘**L**R**Y**PYPSY’ (AmelY;[M+2]$^{2+}$ 529.7689 m/z) that are only present in the enamel of males. We applied this method to eight modern cattle (*Bos taurus*) of known sex, and correctly assigned them to sex. We then applied the same protocol to twelve archaeological *Bos* teeth from the Neolithic site of Beisamoun, Israel (8-th–7-th millennium BC) and determined the sex of the archaeological samples. Since teeth are usually better preserved than bones, this innovative protocol has potential to facilitate sex determination in ancient and modern bovine remains that currently cannot be sexed.

## Introduction

Sexing paleofaunal remains can provide important data on a broad spectrum of issues. These include the animal’s life history, extent of dimorphism, socio-ecological structure and behavior, as well as predator-prey relations and herd management strategies^1–7^. However, sex determination of fossil fauna is severely hampered by the often fragmentary nature of specimens, as it relies on the presence of sex-specific morphological features (e.g. on the pelvis, bacula, horns etc.) that are often missing or broken^8,9^. Additionally, to determine an animal’s sex, researchers have commonly relied on bone and tooth measurements (osteometry), and more recently, on geometric morphometrics^10–15^. However, these methods also rely on the presence of well-preserved remains, while there is often ambivalence in interpreting the mechanism/s responsible for size patterning, since this may be confounded by factors such as climate change, nutrition and domestication^1,16^. While, the advent of aDNA analyses has offered revolutionary possibilities for sexing ancient fauna remains^7,11,13,17^, in many situations its application is limited by DNA degradation and contamination^18^. Clearly, additional tools that will enable sexing of fossil (and fragmentary modern) animal remains will be invaluable to researchers in zoology, archaeology, archaeozoology and paleontology.

To explore new methods of sexing mammals, researchers have turned to the analysis of amelogenin, an essential protein for tooth enamel development in mammals^19–22^. The enamel proteome is composed of around 90% of amelogenin (Amels) dimorphic proteins, comprising the X-linked AmelX that is present in both males and females and the Y-linked AmelY that is only present in males^23–25^. Compared to AmelX, AmelY carries multiple single nucleotide polymorphisms (SNPs) or DNA mutations (e.g. Intron 3, 6 and Exon 5 regions), which translate into single amino acid variations (SAAVs). Thus, the identification of the unique AmelY peptides distinguishes males from females based on relative peptide abundance, and enables the unambiguous identification of males. Numerous studies have used this concept to determine the sex of recent animals by sampling their soft tissues and blood^26–35^. This approach also served as the foundation of groundbreaking sex determination in humans first undertaken by Stewart et al.^36,37^ and Parker et al.^38^ through the analysis of native peptides in the tooth enamel proteome using mass spectrometry techniques. Native peptides in enamel are the result of in-vivo enamel protease digestion, such as matrix-metalloproteinase 20 (Mmp20) and kallikrein-related peptidase 4 (Klk4) enzymes^22,39,40^. The extraction of the native amelogenin peptides facilitates rapid analysis, without the need for enzymatic digestion that accompanies bottom-up proteomic pipelines that may contribute to sample loss in low abundance samples^41^.

This approach has opened the door to sex identification of ancient remains by focusing on tooth enamel, the most resistant material in the skeleton, thereby overcoming the limitations imposed by poor bone preservation commonly encountered in aDNA and other standard sex determination methods outlined above. Not surprisingly, proteomic analyses of enamel has been extensively applied to sex ancient human populations including infants and ancient primates^42–44,44–54^.

Far fewer studies have been undertaken on tooth enamel of fossil fauna. An exception is the study by Cappellini et al.^55^ who successfully identified both the sex and species of fossil rhinoceros remains from the site of Dmanisi, dating to 1.77–1.9 Mya. As a control, they used a male Medieval caprine’s raw spectral data and the AmelY sequence from modern sheep as a matching database. The authors exploited single amino acid variances (SAAVs) in the AmelY sequence at position 171-where valine (V) replaces methionine (M), found in the corresponding position of the AmelX sequence. This key distinction enabled the sex identification of ancient faunal remains from this site.

In the current study, we demonstrate that Label Free Quantification (LFQ) of native peptides extracted from teeth of modern bovine samples permits confident identification of sex. This method was then successfully applied to twelve archaeological samples from a Neolithic site dated to the 8th–7th millennia BC, using the same selected peptides. To our knowledge, this is the first application of a method to determine an animal’s sex using bovine tooth enamel.

## Results

### Sex determination in modern and ancient bovine enamel

**Figure 1:**
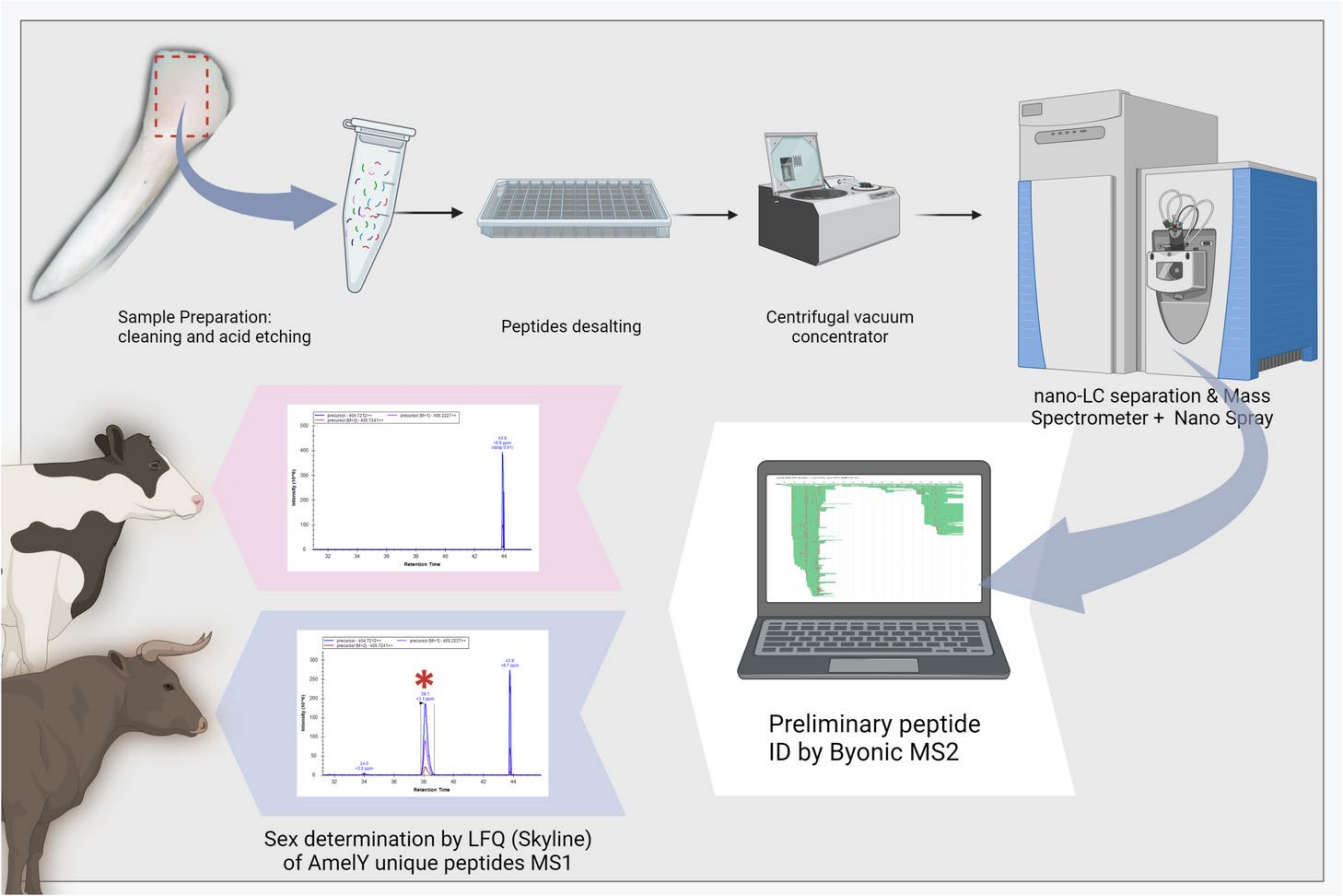
Proteomic workflow for sex determination in modern and archaeological tooth samples. Teeth were cleaned and etched in acid. Acid-dissolved peptides were desalted and run on LC-MS instruments using the DDA method. The data search was undertaken using a Byonic search engine and quantified with Skyline. For greater details see Methods.

**Figure 2:**
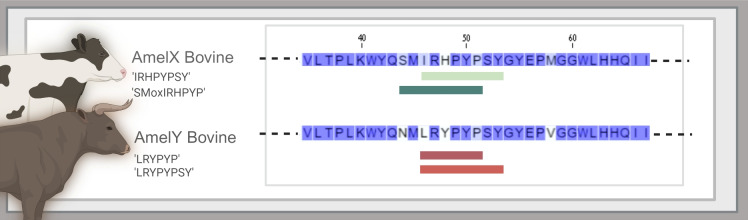
To illustrate the unique sequence of the selected peptides, we aligned AmelY and AmelX sequences of cattle with our selected AmelY and AmelX peptide sequences (red, green lines respectively) as follows: ‘**L**R**Y**PYP’ (AmelY;[M+2]$^{2+}$ 404.7212 m/z), ‘**L**R**Y**PYPSY’ (AmelY;[M+2]$^{2+}$ 529.7689 m/z); AmelX peptide sequences (green lines): ‘**S**M(ox)**I**R**H**PYP’ (AmelX;[M+2]$^{2+}$ 508.7527 m/z) and ‘**I**R**H**PYPSY’ (AmelX;[M+2]$^{2+}$ 516.7667 m/z). SAAVs between the dimorphic proteins are show in light blue.

**Figure 3:**
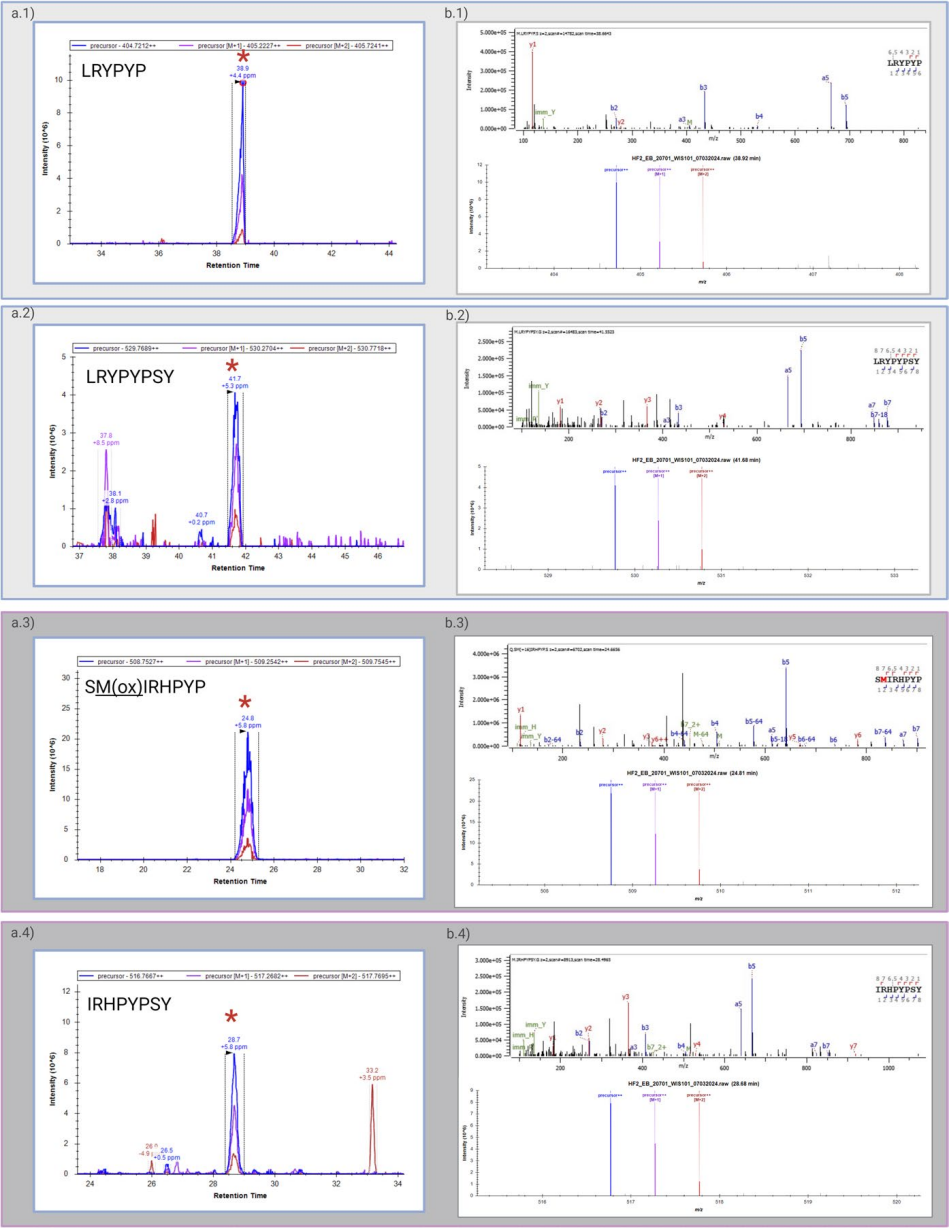
XIC of the isotopic envelope precursor obtained for modern male WIS101 for two unique AmelY and two unique AmelX peptides. (**a.1**) ‘**L**R**Y**PYP’ AmelY;[M+2]$^{2+}$ 404.7212 m/z (**a.2**) ‘**L**R**Y**PYPSY’ AmelY;[M+2]$^{2+}$ 529.7689 m/z; AmelX unique peptides: (**a.3**) ‘**S**M(ox)**I**R**H**PYP’ (AmelX;[M+2]$^{2+}$ 508.7527 m/z and (**a.4**) ‘**I**R**H**PYPSY’ AmelX;[M+2]$^{2+}$ 516.7667 m/z. Retention time was as expected based on a previous Byonic search ID. Figures from (**b.1**) to (**b.4**) illustrate MS2 spectra of the peptide ID, below is the isotopic envelope of the precursor.

**Figure 4:**
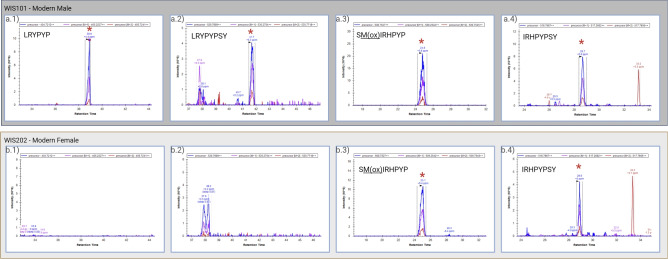
XIC of the isotopic envelope precursor obtained using Skyline software for (**a**) a modern male WIS101 (**b**) a modern female WIS202, for two unique AmelY and two unique AmelX peptides. First, ‘**L**R**Y**PYP’ AmelY;[M+2]$^{+2}$ 404.7212 m/z [(**a.1**) male and (**b.1**) female)], and ‘**L**R**Y**PYPSY’ AmelY;[M+2]$^{+2}$ 529.7689 m/z [(**a.2**) male and (**b.2**) female)]. Second, AmelX unique peptides: ‘**S**M(ox)**I**R**H**PYP’ (AmelX;[M+2]$^{+2}$ 508.7527 m/z [(**a.3**) male and (**b.3**) female)] and ‘**I**R**H**PYPSY’ AmelX;[M+2]$^{+2}$ 516.7667 m/z [(**a.4**) male and (**b.4**) female)]. The retention time was as expected based on a previous Byonic search ID. AmelY peptides were exclusively found in male samples, while female samples lacked the unique AmelY peptides. However, both unique peptides of AmelX were found in both sexes.

**Figure 5:**
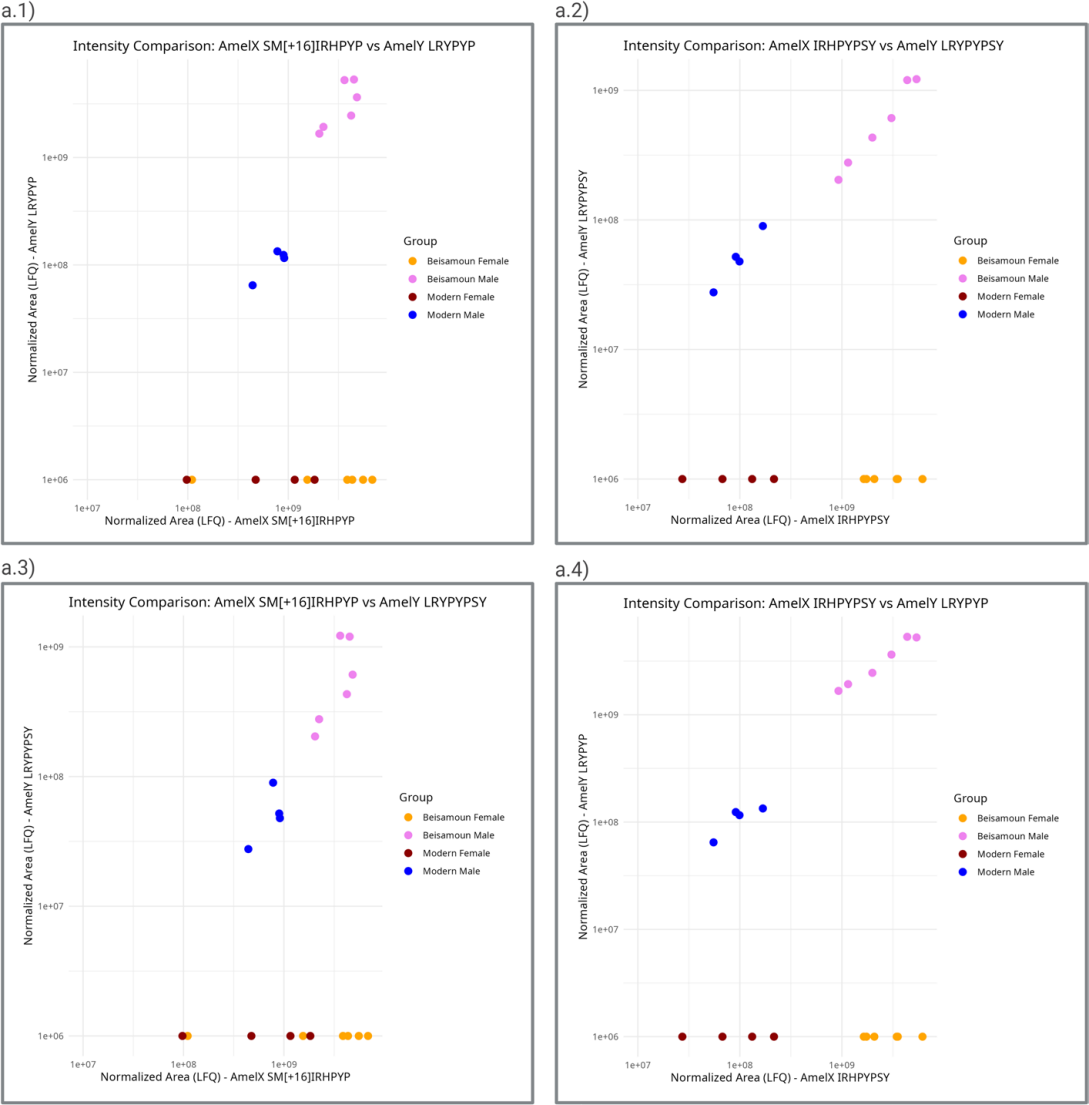
Ion intensities of unique AmelY peptides vs AmelX unique peptide intensities, (**a.1**) ‘**L**R**Y**PYP’ (AmelY;[M+2]2+ 404.7212 m/z) vs ‘**S**Mox**I**R**H**PYP’ (AmelX;[M+2]$^{2+}$ 508.7527 m/z); (**a.2**) ‘**L**R**Y**PYPSY’ (AmelY;[M+2]$^{2+}$ m/z 529.7689) vs ‘**S**Mox**I**R**H**PYP’ (AmelX;[M+2]$^{2+}$ 508.7527 m/z); (**a.3**) **S**M(ox)**I**R**H**PYP (AmelY;[M+2]$^{2+}$ 404.7212 m/z) vs ’**I**R**H**PYPSY’ (AmelX;[M+2]$^{2+}$ 516.7667 m/z); (**a.4**) ‘**L**R**Y**PYPSY’ (AmelY;[M+2]$^{2+}$ m/z 529.7689) vs ‘**I**R**H**PYPSY’ (AmelX;[M+2]$^{2+}$ 516.7667 m/z). Modern male: dark blue dots, modern female: bordeaux dots; archaeological samples (Beisamoun) males (identified by this method): violet dots, and archaeological samples (Beisamoun) females: orange dots. Results are for eight modern samples and twelve archaeological samples.

Sex determination can be assessed through the identification and quantification of the unique peptides of AmelY (for  our workflow, refer to Fig. 1). We selected peptides carrying SAAVs: Leu46, Tyr48, namely ‘**L**R**Y**PYP’ (AmelY;[M+2]$^{2+}$ 404.7212 m/z) and ‘**L**R**Y**PYPSY’ (AmelY;[M+2]$^{2+}$ 529.7689 m/z). The sequence alignment of these unique peptides is illustrated in Fig. 2. In addition, to ensure the correct sex determination and quality of our acid enamel extraction, AmelX dimorphic unique peptides were also quantified. We selected peptides carrying SAAVs, Ser 44, Ile46 and His48. AmelX selected peptides were: ‘**S**M(ox)**I**R**H**PYP’(AmelX;[M+2]$^{2+}$ 508.7527 m/z) and ‘**I**R**H**PYPSY’ (AmelX;[M+2]$^{2+}$ 516.7667 m/z). A BLAST search for these sequences revealed that they exclusively exhibit homology with amelogenin proteins (reviewed sequence by Uniprot). The identifier (ID) for each of these peptides was validated manually (example in Fig. 3b) and the isotopic envelope for the associated peak was corrected (example in Fig. 3a). Sex determination was performed by label-free quantification (LFQ) of native dimorphic peptides unique to AmelY. We found modern male samples had an intensity of AmelY unique peptides between XIC 6.47 $10^7$ and 1.34 $10^8$ for the short peptide version and between 2.76 $10^7$ and 8.97 $10^8$ for the long peptide version. For an example of the disparity between the sexes, in Fig. 4 we plotted sample WIS101 (a modern male) and sample WIS202 (a modern female). Figure 5 shows the intensities of modern male (blue dots) and female (red dots) samples. In addition, we calculated the average AmelX deamidation that was between 21% and 16.42% for Asp and Gln respectively.

As proof of concept, we applied the above method to the analysis of archaeological enamel obtained from twelve bovine teeth from the Neolithic (8th–7-th millennia BC) site of Beisamoun (for details on the sites see Materials section below). We were able to determine sex in all archaeological samples. AmelY unique peptides identified six males out of twelve archaeological samples from Beisamoun at XIC $2.76 \times 10^7$ to $5.33 \times 10^9$ (see Fig. 5violet dots). In addition, AmelX unique peptides were found across all archaeological samples at XIC between $1.10 \times 10^8$ and $6.87 \times 10^9$ (see Fig. 5, red dots). Sex determination was possible due to the presence of AmelX unique peptides, and the presence or lack of AmelY. See also SI 1 and SI 2.

Global deamidation was used as a tool to validate antiquity and study diagenesis in the archaeological samples^56–58^. We calculated Asp and Gln bulk deamidation of AmelX and compared the modern and archaeological samples. Archaeological samples showed 3 and 5 fold higher occupancy of deamidation (Asp and Gln, respectively) compared to modern samples, validating the ancient origin of our fossil samples.

## Discussion

We have presented here, a method for sex determination in bovine enamel based on LFQ of peptides unique to AmelY. We selected two peptides unique to AmelY (’**L**R**Y**PYP’ and the ’**L**R**Y**PYPSY’) that are found exclusively in male bovines. We also found two peptides unique to AmelX (‘**S**Mox**I**R**H**PYP’ and ‘**I**R**H**PYPSY’). All four peptides re found in both modern and archaeological samples in abundance, allowing reliable quantification and thereby robust sex determination. The use of peptidomics is advantageous, as it simplifies sample preparation and reduces the introduction of sample preparation artefacts, while still providing the desired methodological fidelity.

## Conclusions

Our method provides a simple and reliable method for sex determination in bovines using dental enamel of modern and archaeological samples, through the application of LFQ peptidomics. It will be of particular use for sexing poorly preserved osteological samples, those lacking diagnostic morphology, or assemblages where DNA methods cannot be applied due to poor preservation.

### Ethics statement

The modern cattle samples used in this study derive from animals that were sold for slaughter to an authorized slaughterhouse (Beit Mitbahaim Tira Ltd.) and were not intentionally killed for this study. Lower jaws used in this study are routinely discarded by the slaughterhouse as they have no commercial value. The Neolithic site of Beisamoun was excavated under permit from the Israel Antiquities Authority (granted to co-authors FB & HK). For details on the site, its location and excavations, see section on Archaeological samples below. Permission to sample bovine teeth was obtained from the excavators as well as from the archaeozoologist (LKH) working on this assemblage (all of whom are co-authors on this paper). The collection is currently held in the laboratory of the latter researcher, in the National Natural History Collections of the Hebrew University of Jerusalem.

## Methods

### Modern samples

A total of eight modern cattle lower jaws were donated by an Israeli meat manufacturer, Beit Mitbahaim Tira Ltd. (see Table 1). For photographs of samples see SI 3.

**Table 1:** The table summarizes modern sample information including: Sample number, Proteomic Batch, Animal ID, Animal Species, Sex, Tooth type (M=molar; PM=premolar; I=incisor).

Sample number	Proteomic batch	Animal ID	Species	Sex by docs	Tooth type
WIS101	20701	Bostaurus10	Bos taurus	Male	M3
WIS201	20701	Bostaurus16	Bos taurus	Female	I3
WIS202	20701	Bostaurus14	Bos taurus	Female	M2
WIS203	20701	Bostaurus12	Bos taurus	Male	I2
WIS204	20701	Bostaurus11	Bos taurus	Male	M2
WIS208	20701	Bostaurus17	Bos taurus	Female	PM3
WIS209	20701	Bostaurus15	Bos taurus	Female	I3
WIS210	20701	Bostaurus13	Bos taurus	Male	M2

### Archaeological samples

The 12 archaeological teeth samples analyzed derive from the Neolithic site of Beisamoun (see Table 2, map ref NIG 25403-8/77682): The Neolithic site of Beisamoun is located in the Upper Jordan Valley, within the flood-plain of the now extinct Hula Lake, a region that is characterised by a Mediterranean climate^59^. The locality was first excavated in the 1960s–1971 by Monique Lechevallier who reported finding remnants of a mid-Pre-Pottery Neolithic B village (PPNB, first half of the 8th millennium BC; Lechevallier 1978)^60^. Renewed excavations in 2007 through 2016, in an adjacent area were undertaken by a Franco-Israeli mission. They exposed in situ layers dating to the late PPNB (second half of the 8th millennium BC) and Pre-Pottery Neolithic C (PPNC, first half of the 7th millennium BC). The finds included architectural remains and built installations, with clearly identifiable successive phases of building and their associated lithic artefacts (e.g. arrowheads, sickle blades, axes, adzes, burins, scrapers, retouched blades etc.), groundstone artefacts (grinding slabs, mortars, hand stones, vessels etc.) predominantly made on basalt and limestone, bone tools, shell, stone and bone ornaments, as well as human burials and faunal remains^61,62^. The PPNC sediment is composed of a sandy-clay sediment with fine-grained inclusions of charcoal, clay, and ochre^61^. For photographs of samples, see SI 3.

**Table 2:** The table summarizes archaeological sample information including: Sample Number, Sample Locus and Square in the archaeological site of Beisamoun, Animal Species, Sex by Amelogenin (this research study), Tooth type  (M=molar; PM=premolar; I=incisor; U=upper; L=lower), Period  (PPNC=Pre-Pottery Neolithic C; PPNB=Pre-Pottery Neolithic B).

Samples number	Proteomic batch	Loc. Sq. (Location Square)	Species	Sex by amels	Tooth type	Period
WIS116	20701	BN14, Area E, Loc. 334, sq. T7b/T8a/U7c/U8d, Numb. 3316	*Bos*	Male	LM1-right	PPNC
WIS115	20701	BN14, Area E, sq: T7c/d, Numb. 3291	*Bos*	Male	UPM3-left	PPNC
WIS164	20701	BN13, Area E, Loc. 371, sq. S7a / T7d, Numb. 3174	*Bos*	Female	UM3 right	PPNC
WIS111	20701	BN12, Area E, sq:R9d; Loc. 339, Numb. 2877	*Bos*	Male	LM2	PPNC
WIS100	20701	BN12, Area E, Loc. 333, sq: S10d/R10b, Numb. 2727	*Bos*	Male	PM4-right	PPNC
WIS114	20701	BN14, Area E, Loc. 352, Numb. 3391, sq: U7a-d	*Bos*	Female	LM1 left	PPNC
WIS102	20701	BN10, Area F, Sq. W25a; Numb.1827	*Bos*	Female	LM	PPNB-PPNC
WIS110	20701	BN 11, Area F, Loc. 228, sq: V24c; Numb. 5034	*Bos*	Female	UM-1/2	PPNB-PPNC
WIS165	20701	BN16, Area E, sq. S7 a/d, Numb. 3907	*Bos*	Female	UM1 right	PPNB-PPNC
WIS166	20701	BN11, Area F, Numb. 5045, sq. T21b, Loc 243/246	*Bos*	Female	LM1 right	PPNB-PPNC
WIS163	20701	BN10, Area F, q. U25d, Numb. 1833	*Bos*	Male	UM(?)	PPNB-PPNC
WIS109	20701	BN 11, Area F, Numb. 5021 sq. U26c	*Bos*	Male	UM-1/2-left	PPNB-PPNC

### Samples preparation and cleaning

Teeth (modern and archaeological) were first cleaned mechanically using soft brushes to exclude any external material (e.g. sediments). Using bistoury and tweezers under a microscope binocular (Nikon SMZ800N, see picture SI 6),  a clean piece of enamel (10 mm $\times$ 15 mm) was extracted. A final examination was carried out using a Nikon SMZ800N binocular microscope. Finally, etching was performed under a chemical hood (see subsection, Sample Preparation for MS).

### Sample preparation for MS

The extracted and clean piece of bovine enamel was immersed for 30 s in 3% $\text {H}_{2}\text {O}_{2}$, rinsed with DDW and the solution discarded. Then, the enamel piece wasmanually ground and etched for 2 min in freshly prepared 5% HCl. This solution was discarded. A second etching step in 1000 $\upmu$l 5% HCl for 60 min was performed at room temperature, and the solution was retained in a new Eppendorf on ice. The piece of enamel was then allowed to completely dissolve in 1000 $\upmu$l 5% HCl (aprox. 90 min). Then, both solutions were combined to a final volume of 2000 $\upmu$l (about 100 mg dry weight). The samples were then frozen and stored in $-80\;{^circ}$C for desalting.

### Mass spectrometry

Dissolved samples were desalted using Oasis HLB 96 well plate (Waters), using the manufacturer’s instructions; samples were loaded into the wells using vacuum pull, followed by three washes of 300ul 0.1% TFA. The peptides were then eluted by passing 50μl 50% Acetonitrile, 0.1% FA. The resulting peptides were loaded into a reversed-phase Symmetry C18 trapping column (20*0.18 mm, 5  $\upmu$m particle, Waters) and resolved on an analytical HSS T3 column (250*0.07 5mm, 1.8  $\upmu$m particles, Waters), mounted on a nanoAquity (Waters) nanoLC instrument. Peptides were eluted from the column using a 50-minute gradient from 4 to 30%B (99.9% acetonitrile, 0.1% formic acid) at 350 nl/min flow. The peptides were eluted into a Q-Exactive HF mass spectrometer (Thermo Fisher) using a FlexIon nano-ESI source, through a 20  $\upmu$m ID emitter (Fossil IonTech, Madrid), at 2.8 kV. Blank injections interspaced samples to allow washing off peptide carryover. Data were acquired in data-dependent acquisition (DDA) mode using Top10 method. MS1 resolution was set to 120,000@ 400 m/z, with a mass range of 350–1650 m/z, using a maximum injection time of 60 ms. Precursors selected for MS2 were limited to charge states of 2–8, intensity threshold of $3.3^4$ and dynamic exclusion of 20s. Precursors were isolated using a window of 1.7 m/z, AGC target was set to $10^5$ or a maximum of 60ms injection time and fragmented using HCD at 27 normalized collision energy (NCE). Scans were performed using a first mass setting of 100 m/z at 15,000 resolution (@200 m/z).

#### Data analysis

Data was searched using a Byonic search engine (Protein Metrics Inc) with a database that was tailored for modern and ancient samples against the relevant sequences (P02817 AMELX BOVIN Amelogenin, X isoform, *Bos taurus* and Q99004 AMELY BOVIN Amelogenin, Y isoform *Bos taurus*, Uniprot). Fixed and variable modifications were adjusted as follows: common2: Oxidation (F, H, K, M, P, W, Y), Deamidated (N, Q, R); rare1: Acetyl (NTerm), Gln to pyro-Glu (NTerm Q), Glu to pyro-Glu (NTerm E) and Phosphorilation (S); rare2: Di-oxidation (F, M, P, W, Y). maximum of 2 common and 2 rare modifications were allowed. MS1 tolerance was set to 10ppm while MS2 was set to 20ppm. Data was filtered using Byonic’s target-decoy method set to 1 % percent FDR. We used a post-search cut-off of LogProb $>3$ (FDR $< 0.001$) and score $>200$. Identified unique AmelY peptides were examined manually to avoid false positive identifications. Quantification was performed using Skyline software (version 23.1.0.455). Byonic mzID files were imported into Skyline along with the RAW files and used to identify the correct peak for quantification positively. Quantification was performed by XIC extraction of the MS1 signal. Quantification of AmelY base on peptide1: ‘**L**R**Y**PYP’ (AmelY;[M+2]$^{2+}$ 404.7212 m/z); peptode2:‘**L**R**Y**PYPSY’ (AmelY;[M+2]$^{2+}$ 529.7689 m/z). AmelX: peptide3:‘**S**Mox**I**R**H**PYP’ (AmelX;[M+2]$^{2+}$ 508.7527 m/z); peptide4:‘**I**R**H**PYPSY’ (AmelX;[M+2]$^{2+}$ 516.7667 m/z). Ion fragment validation was performed manually. We performed sequence similarity searches using protein-protein BLAST program (www.uniprot.org/blast). Bulk modification calculations were performed by in-house RStudio scrips.

### Supplementary Information

Supplementary Information 1.

Supplementary Information 2.

## Data Availability

The mass spectrometry proteomics data have been deposited to the ProteomeXchange Consortium via the PRIDE^63^ partner repository with following dataset identifiers: (1) PXD053146, 10.6019/PXD053146; (2) PXD053148, 10.6019/PXD053148. LFQ files at PXD053225. ProteomeXchange via the PRIDE database-Password and user names for reviewers: The data was divided into 3 projects, see all the details below. a) Project Name: Label-Free Quantification Method for Assessing Sex from Modern and Ancient Bovine Tooth Enamel. • Project accession: PXD053146 • Project DOI: 10.6019/PXD053146 b) Project Name: Label-Free Quantification Method for Assessing Sex from Modern and Ancient Bovine Tooth Enamel PART II. • Project accession: PXD0531486 • Project DOI: 10.6019/PXD053148 c) Project Name: Label-Free Quantification Method for Assessing Sex from Modern and Ancient Bovine Tooth Enamel. • Project accession: PXD053225.
