# Supplementary material for: A label-free quantification method for assessing sex from modern and ancient bovine tooth enamel
Source: Sci Rep. 2024 Aug 6;14:18195. doi: 10.1038/s41598-024-68603-4 (PMC11303769; doi:10.1038/s41598-024-68603-4)

Supplementary Information

Kotli et al. Label-Free Quantification Method for Assessing Sex from Modern and Ancient Bovine Tooth Enamel.

SI 1. Archeological sample male WIS109.

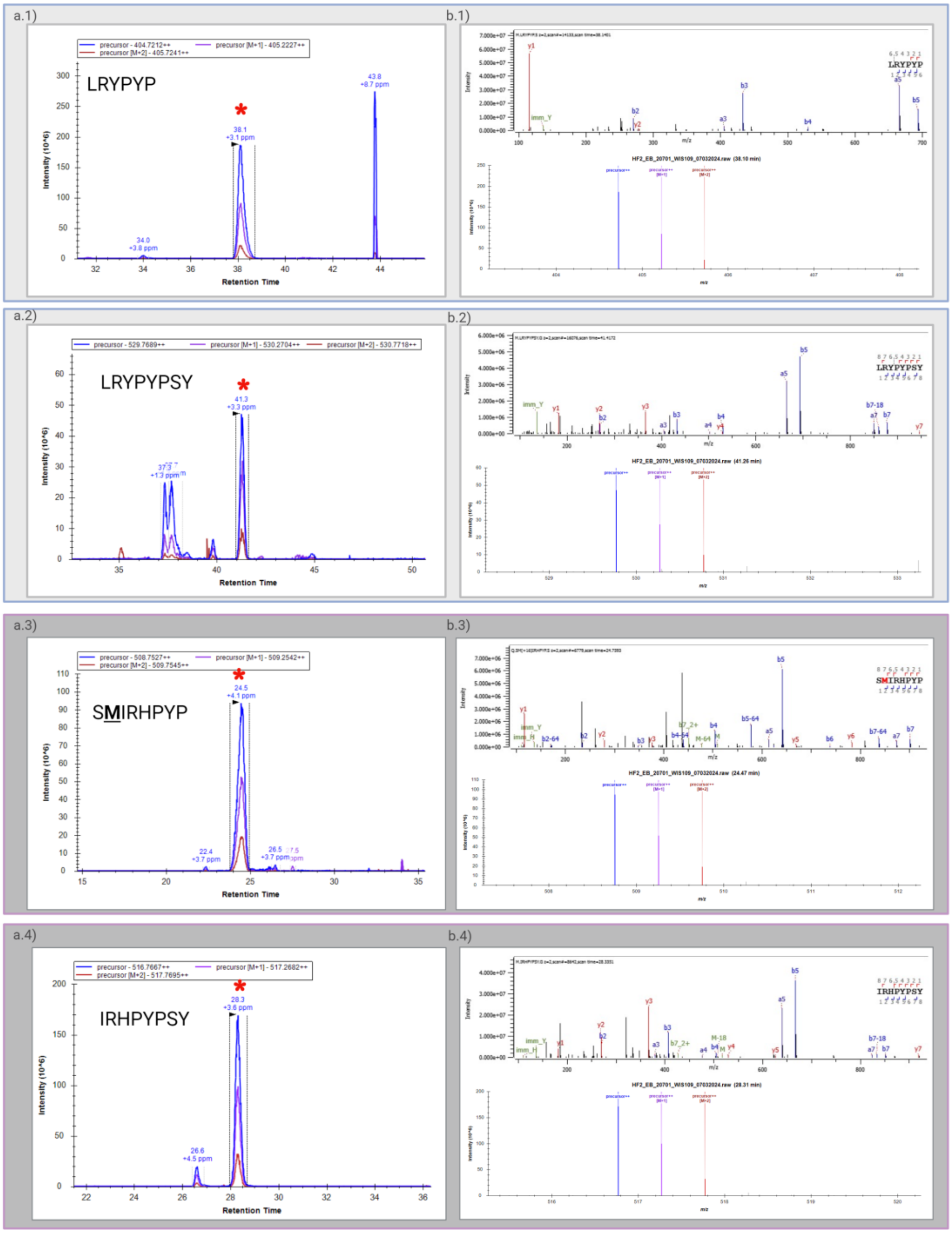

Fig. 1. XIC of the isotopic envelope precursor obtained for archeological samples male WIS109 of two unique AmelY and two unique AmelX peptides. a.1)‘LRYPYP’ AmelY;[M+2]2+ 404.7212 m/z a.2)‘LRYPYPSY’ AmelY;[M+2]2+ 529.7689 m/z; AmelX

unique peptides: a.3) 'SM(ox)IRHPYP' (AmelX;[M+2]2+ 508.7527 m/z and a.4) 'IRHPYPSY' AmelX;[M+2]2+ 516.7667 m/z. RT were as expected by Byonic previous search ID. Figures from b.1) to b.4) correspond MS2 spectra of the peptide ID, below, isotopic envelope of the precursor.

SI 2: Archeologicaal samples: WIS109 (male), WIS110 (female) peptide abundance disparity between the sexes.

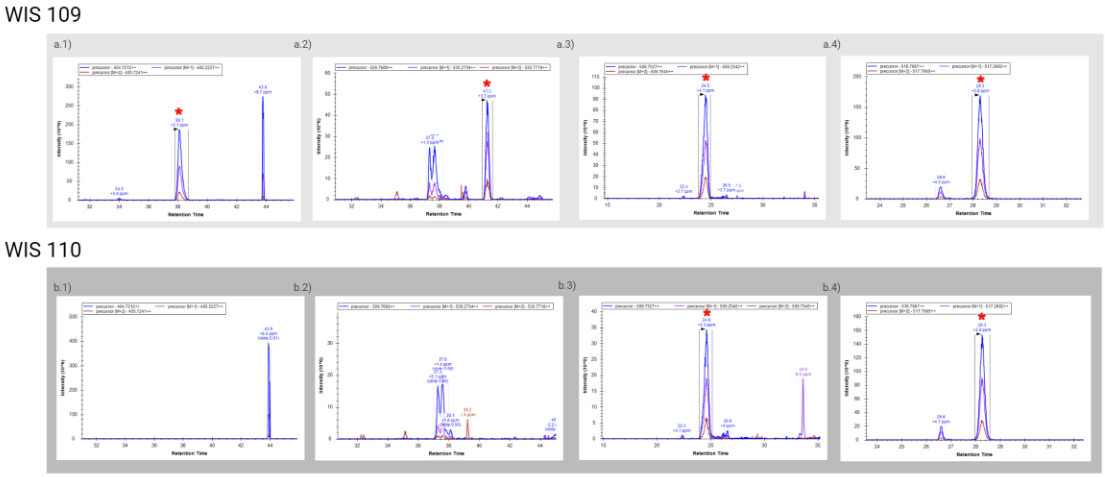

Figure 2. XIC of the isotopic envelope precursor obtained using Skyline software for archeological samples male WIS109 (a) and female WIS110 (b) of two unique AmelY and two unique AmelX peptides. First, 'LRYPYP' AmelY;[M+2]2+ 404.7212 m/z (a.1 -male and b.1 -female), and 'LRYPYPSY' AmelY;[M+2]2+ 529.7689 m/z(a.2 -male and b.2 -female). Second, AmelX unique peptides: 'SM(ox)IRHPYP' (AmelX;[M+2]2+ 508.7527 m/z (a.3 -male and b.3 -female) and 'IRHPYPSY' AmelX;[M+2]2+ 516.7667 m/z (a.4 -male and b.4 -female) the RT were as expected by Byonic previous search ID. AmelY peptides were exclusively found in male samples, where female samples lack the unique AmelY peptides. However, both unique peptides of AmelX were found across all samples sexes.

SI 3: Photos of modern and Neolithic teeth sampling procedure

Modern cattle (Bos taurus), In total 8 samples (4 Females, 4 Males), obtained from the abattoir Tira Ltd., Israel

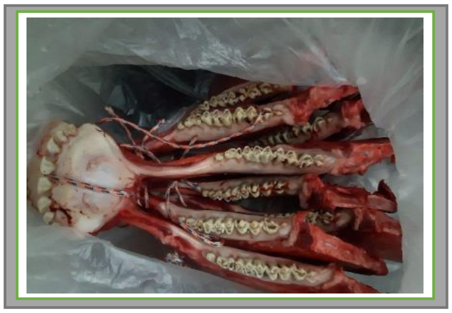

Figure 3 .Four complete male cattle mandibles photographed when they were first received from the abattoir.

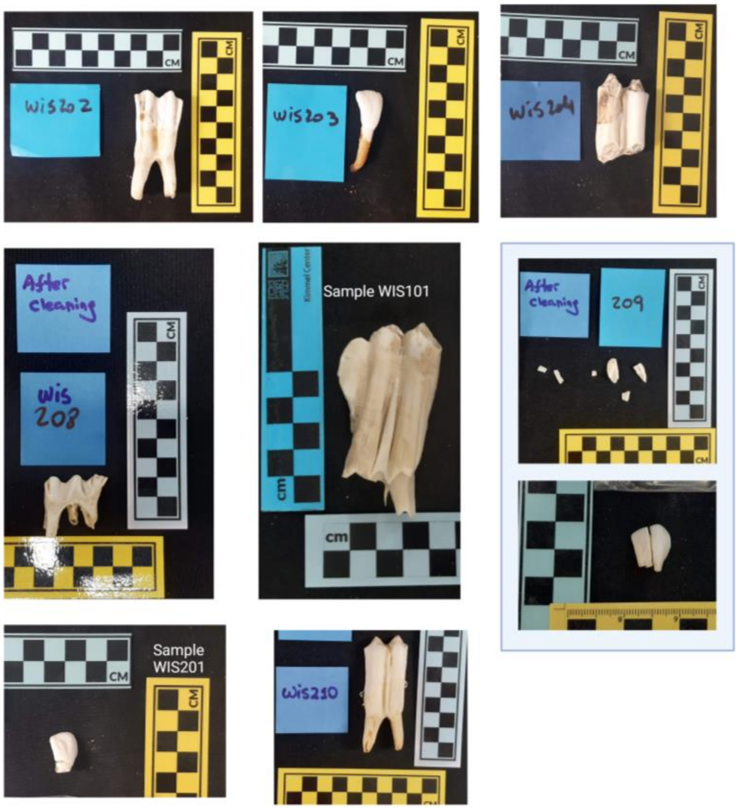

Figure 4. *Modern samples used in this research work, 4 males and 4 female samples.*

Archeological Samples – Beisamoun

Below, *Beisamoun, Neolithic period, Bos sp. samples of unknown sex.* The samples were enclosed in a “cement” matrix that was only possible to remove with mechanical tools. For more details refer to the “Methods” section.

SAMPLE WIS100

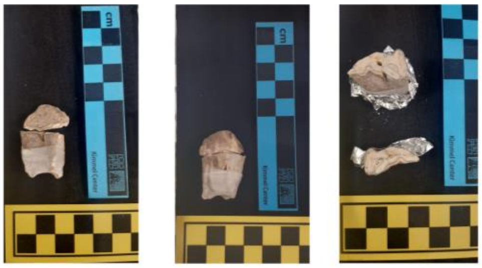

SAMPLE WIS102

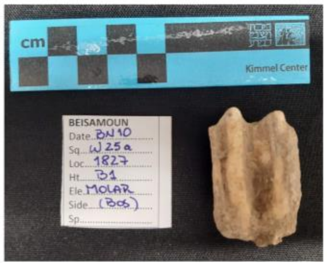

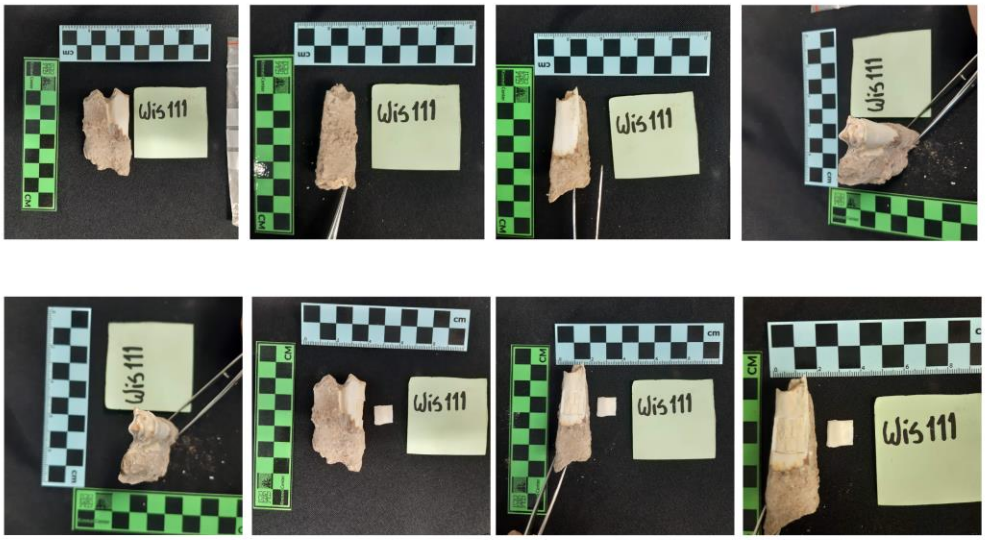

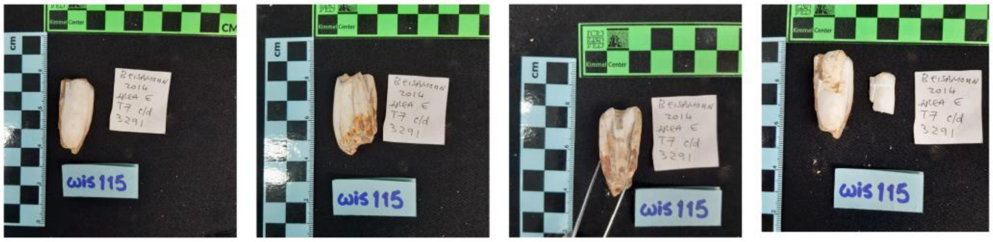

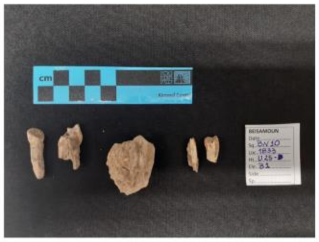
cm
BEISAMOUN

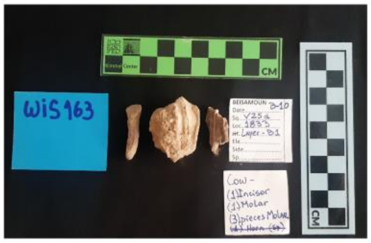
WiS963
BEISAMOUN
CM
Cow -
(1)Incisor
(1)Molar
(3)pieces Molar

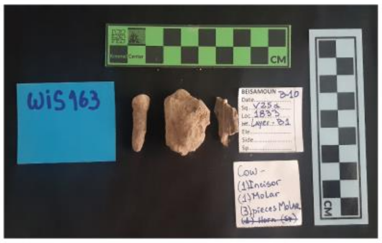
WiS963
BEISAMOUN
CM
Cow -
(1)Incisor
(1)Molar
(3)pieces Molar

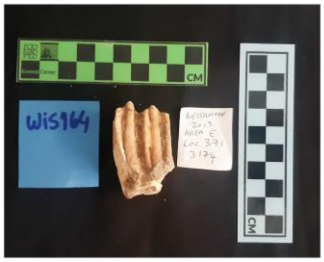
WiS964
CM

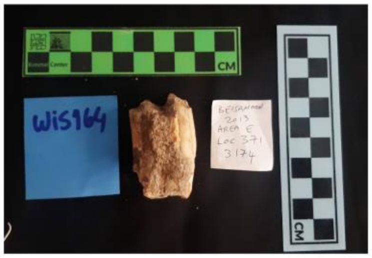
WiS964
CM

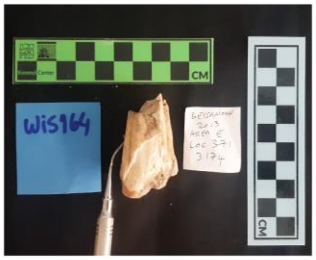
WiS964
CM

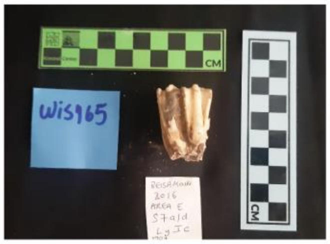
WiS965
CM

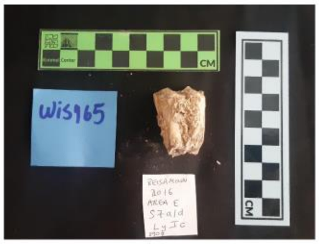
WiS965
CM

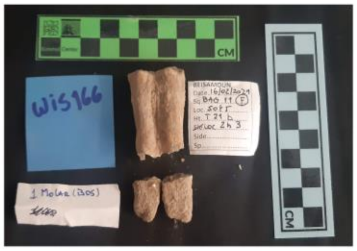
WiS966
BEISAMOUN
CM
1 Molar (Bos)

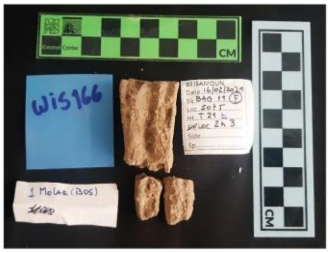
WiS966
BEISAMOUN
CM
1 Molar (Bos)

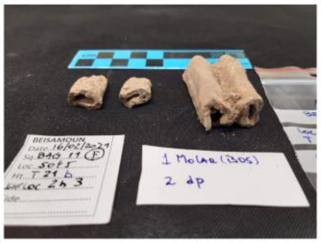
BEISAMOUN
1 Molar (Bos)
2 dp

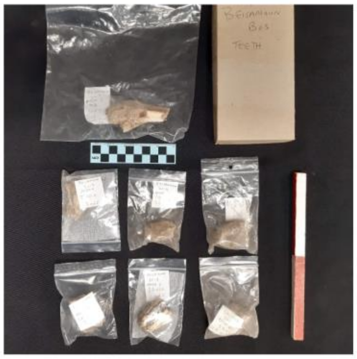

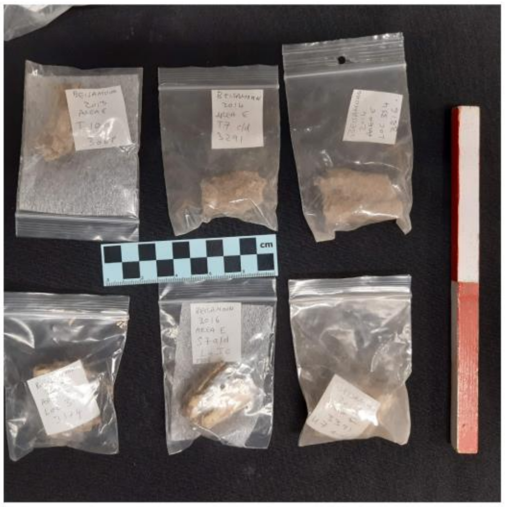

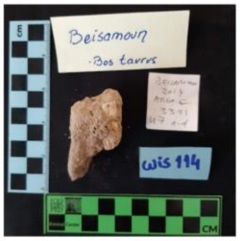
cm
Beisamoun
·Bos taurus
wis 114
Kimmel Center
CM

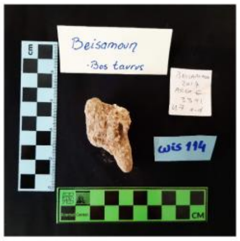
Beisamoun
·Bos taurus
wis 114
Kimmel Center
CM

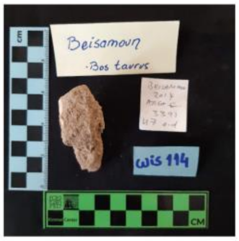
cm
Beisamoun
·Bos taurus
wis 114
Kimmel Center
CM

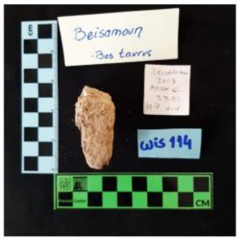
cm
Beisamoun
·Bos taurus
wis 114
Kimmel Center
CM

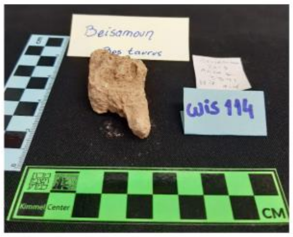
cm
Beisamoun
·Bos taurus
wis 114
Kimmel Center
CM

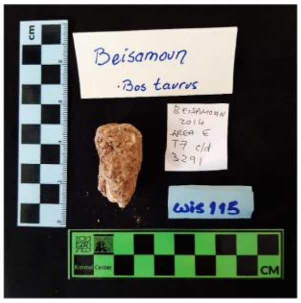
cm
Beisamoun
·Bos taurus
BEISAMOUN
2016
AREA E
T7 c/d
3291
wis 115
Kimmel Center
CM

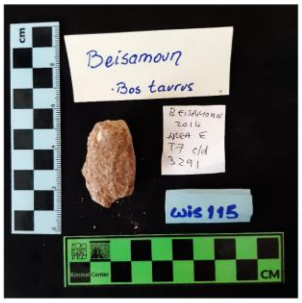
cm
Beisamoun
·Bos taurus
BEISAMOUN
2016
AREA E
T7 c/d
3291
wis 115
Kimmel Center
CM

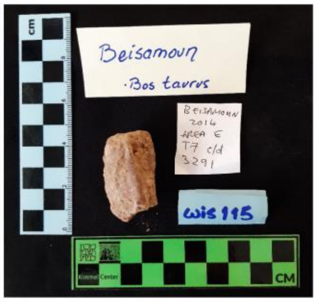
cm
Beisamoun
·Bos taurus
BEISAMOUN
2016
AREA E
T7 c/d
3291
wis 115
Kimmel Center
CM

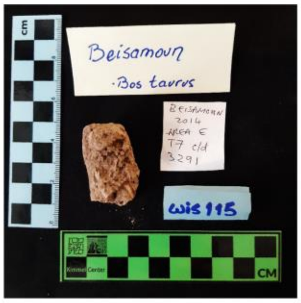
cm
Beisamoun
·Bos taurus
BEISAMOUN
2016
AREA E
T7 c/d
3291
wis 115
Kimmel Center
CM

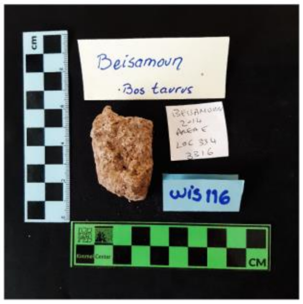
cm
Beisamoun
·Bos taurus
BEISAMOUN
2016
AREA E
LOC 334
3316
wis 116
Kimmel Center
CM

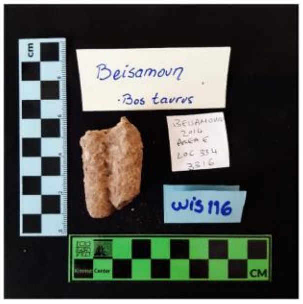
cm
Beisamoun
·Bos taurus
BEISAMOUN
2016
AREA E
LOC 334
3316
wis 116
Kimmel Center
CM

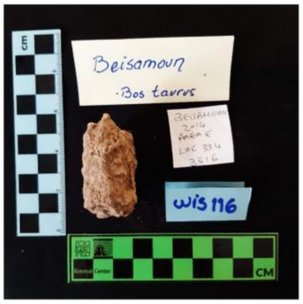
cm
Beisamoun
·Bos taurus
BEISAMOUN
2016
AREA E
LOC 334
3316
wis 116
Kimmel Center
CM

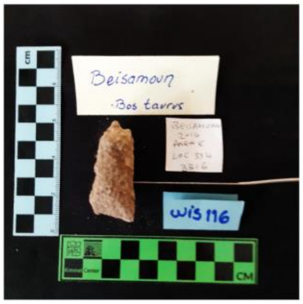
cm
Beisamoun
·Bos taurus
wis 116
Kimmel Center
CM

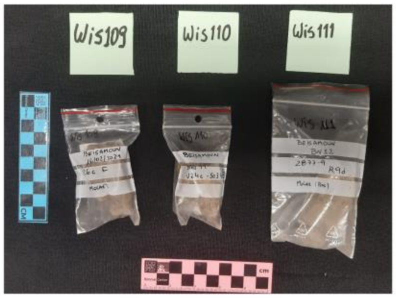
Wis109
Wis110
Wis111

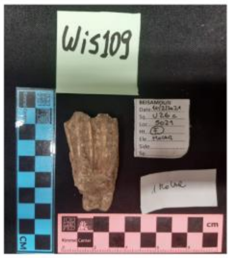
Wis109

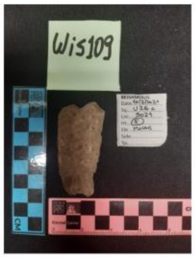
Wis109

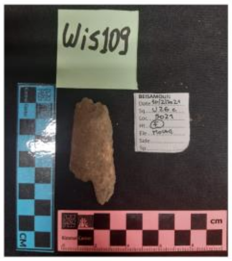
Wis109

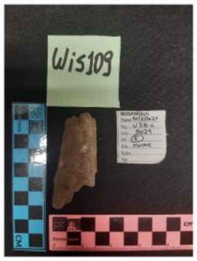
Wis109

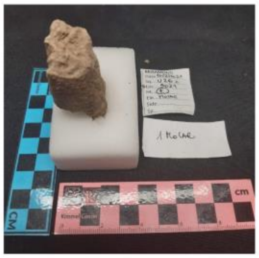

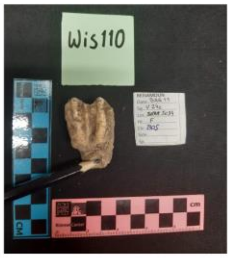
Wis110

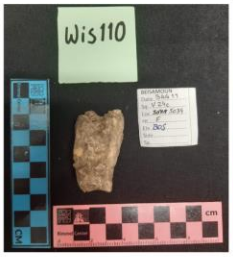
Wis110

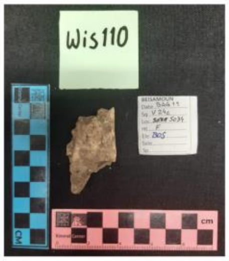
Wis110

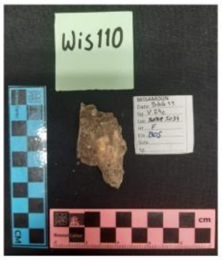
Wis110

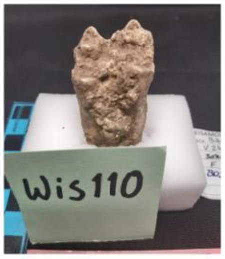
Wis110

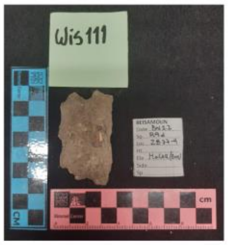
Wis111

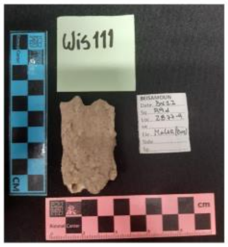
Wis111

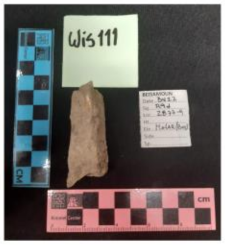
Wis111

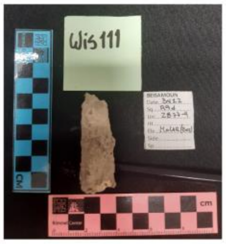
Wis111

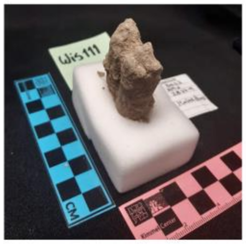

Supplement: Supplementary file 1 — Supplementary Information 1. [file 41598_2024_68603_MOESM1_ESM.pdf]
